# Transcriptomic sequencing and differential analysis of Kazakh horse muscles from various anatomical locations

**DOI:** 10.3389/fvets.2025.1633786

**Published:** 2025-07-24

**Authors:** Mierkadina· Wubulikasimu, Jiahao Liu, Xinkui Yao, Jun Meng, Jianwen Wang, Yaqi Zeng, Linling Li, Wanlu Ren

**Affiliations:** ^1^College of Animal Science, Xinjiang Agricultural University, Urumqi, China; ^2^Xinjiang Key Laboratory of Equine Breeding and Exercise Physiology, Xinjiang Agricultural University, Urumqi, China

**Keywords:** Kazakh horse, skeletal muscle, transcriptome, mRNA, differentially expressed gene

## Abstract

Kazakh horses, a distinguished breed in China known for their dual-purpose use in milk and meat production, exhibit early maturation, tolerance to coarse feeding, and strong resistance to environmental stress. However, the gene expression differences across various muscle tissues of Kazakh horses have yet to be elucidated. In this study, transcriptomic sequencing was performed on muscle tissues from three anatomical regions of Kazakh horses, including the longissimus dorsi (Gb), external oblique (Gf), and diaphragm (Gg) muscles. In the Gb and Gf groups, 426 differentially expressed genes (DEGs) were identified, including *TPM1, TNNI2, ACTN3*, and *MYH8*, of which 147 were upregulated and 279 downregulated. In the Gf and Gg groups, 1,762 DEGs were detected, including *MYBPH, SLC39A8, EMX2*, and *GRB7*, with 1,391 upregulated and 371 downregulated. Additionally, 644 DEGs were identified between the Gg and Gb groups, including *HOXD9, TBX1, LDHA*, and *PKM*, with 172 upregulated and 472 downregulated. GO annotation and KEGG enrichment analysis revealed that the DEGs, such as *TPM1, TNNI2, ACTN3*, and *MYH8*, were primarily involved in System Development, Extracellular Space, and Protein-Arginine Deiminase Activity. Furthermore, pathways related to skeletal muscle growth, including Cytoskeleton in Muscle Cells, Cytokine-Cytokine Receptor Interaction, and Motor Proteins, were significantly enriched. RT-qPCR analysis validated the accuracy of the transcriptomic sequencing data. This study provides valuable insights into the differential expression of genes and related signaling pathways in various muscle tissues of Kazakh horses, rendering a theoretical foundation and data references for understanding skeletal muscle growth and improving meat production in equines.

## 1 Introduction

Horses have long held a crucial place in human society, contributing to competitive sports, dairy production, and meat supply (1). In China, several horse breeds are widely cultivated, including Kazakh horses, Yili horses, Mongolian horses, and Hequ horses. Among these, Kazakh horses stand out, with a large population and widespread distribution. Known for their stable genetic performance, tolerance to coarse feed, and strong resistance to environmental stress, Kazakh horses are highly valued in breeding programs, recreational riding, and animal product production (2).

Meat products are a pivotal part of the human diet, providing necessary nutrients for growth, development, and overall health. With the global population growth and shifts in dietary habits, the demand for meat has steadily increased (3). Countries with high consumption of horse meat include regions of Central Asia, France, Italy, and Mexico (4). Horse meat is considered a healthier alternative to other meats due to its low fat content, high protein levels, abundant unsaturated fatty acids, and elevated iron content (5–7). Additionally, its high iron content makes horse meat particularly beneficial for treating iron-deficiency anemia in humans (8).

Factors influencing meat quality include tenderness, color, pH value, and muscle fiber type (9, 10). Meat quality is typically assessed through pH, shear force, appearance, water loss rate, drip loss, and cooking loss, alongside chemical composition markers (such as moisture, ash content, protein, and fat) and nutritional value indicators (including amino acids, fatty acids, and minerals) (11, 12). Differences in muscle fiber types are particularly evident across various breeds and muscle regions within a single breed (13), with additional variations attributed to age and gender (14, 15). Skeletal muscle accounts for ~40% of an animal's body weight and is the most abundant tissue in mammals (16). The proportion of muscle tissue is intricately linked to its primary functions (17) and directly impacts the yield and quality of meat in livestock and poultry (18). Skeletal muscle tissues from different anatomical regions exhibit considerable diversity in origin, structure, metabolic traits, and functional capacity. Therefore, understanding the regulatory signaling pathways and differentially expressed genes (DEGs) that modulate muscle development in distinct muscle regions is essential to enhance meat quality and production.

Numerous studies have been conducted on the transcriptomes of skeletal muscle in various animal species, such as cattle (10), sheep (19), and pigs (20). Yu et al. (10) identified three DEGs in skeletal muscle from four specific locations in Qinchuan cattle: *NDUFAB1, NDUFA12*, and *NDUFB7*, which regulate muscle development in different regions and optimize meat quality traits. Transcriptomics and proteomics have provided a theoretical foundation for understanding the molecular mechanisms underlying horse meat quality, with key genes such as *ACTN3, MYOZ2*, and *SLN* being identified as regulators of muscle fiber types, thereby influencing attributes like tenderness and color (21, 22). However, the transcriptomics of various muscle regions in Kazakh horses remains under debate. In response to this gap, this study employed transcriptomic techniques to sequence and analyze muscle samples. The objective is to reveal the biological changes in skeletal muscle development in Kazakh horses, identify DEGs and related signaling pathways in diverse muscle regions, and uncover the key genes and biological processes that modify muscle fiber differentiation. This research provides a foundation for future studies on the mechanisms of skeletal muscle growth and meat production enhancement in equines.

## 2 Materials and methods

### 2.1 Experimental animals

**Figure 1:**
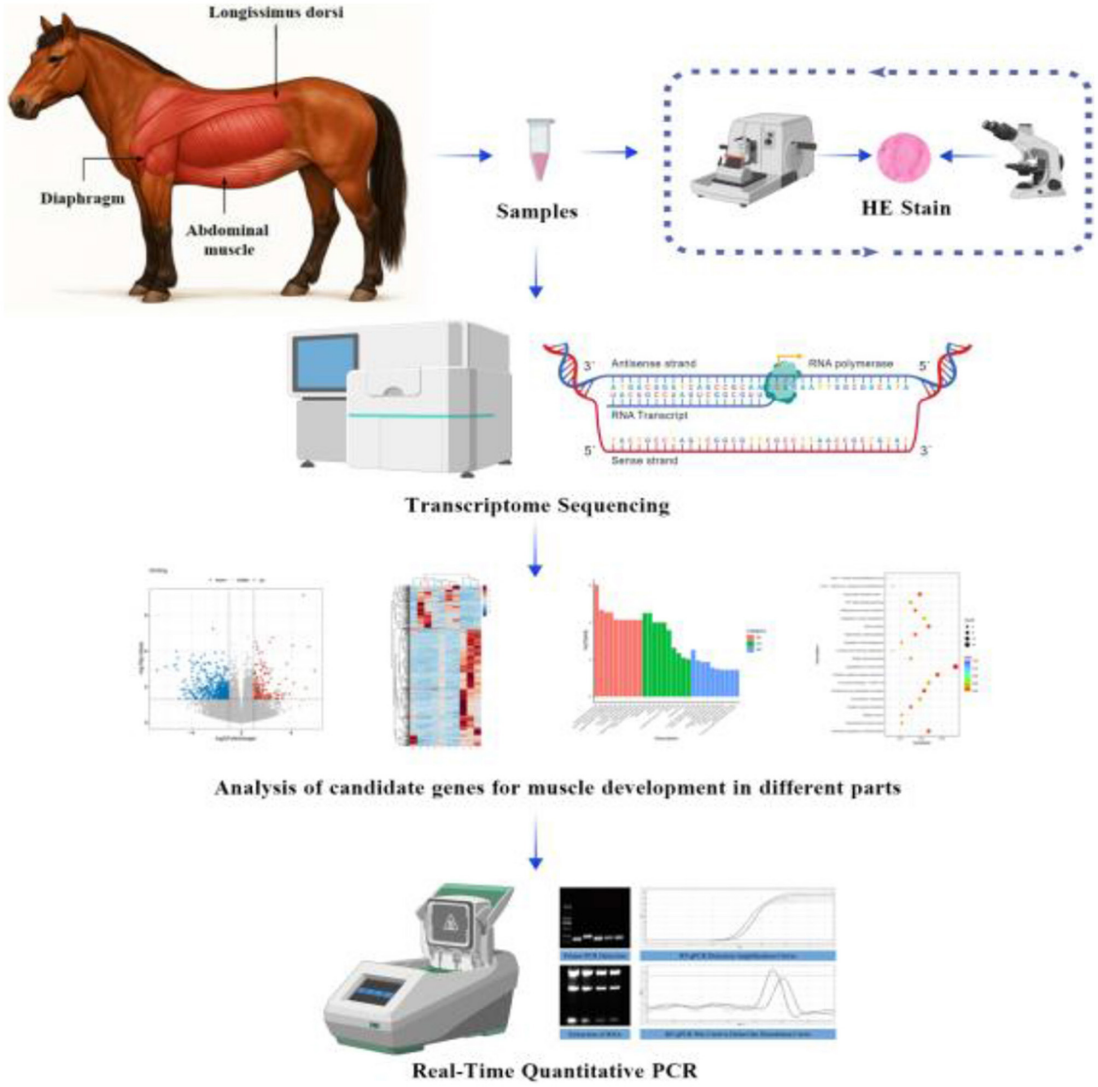
Technology roadmap.

This experiment was conducted in 2025 in the Tacheng prefecture of Xinjiang, China, using five 3-year-old male Kazakh horses. Muscle samples were collected from three muscle groups: the longissimus dorsi (Gb), the external oblique (Gf), and the diaphragm (Gg), with five biological replicates per group. All experimental horses were fed high quality dry alfalfa and corn kernels and unrestricted water under the same husbandry conditions. Figure 1 shows the technical roadmap for this trial. The tissue samples were preserved in liquid nitrogen and 4% paraformaldehyde solution for further analysis. Some samples were stained for HE to make sections, and the other part was subjected to transcriptome sequencing. Drawing is done by bioGDP.com operations.

### 2.2 Micromorphology examination

The muscle tissue samples were fixed in 10% neutral-buffered formalin, dehydrated through a series of alcohol solutions, cleared with xylene, and embedded in paraffin. Tissue sections with a thickness of 4–5 μm were prepared and stained with hematoxylin and eosin (H&E). Images were captured using an optical microscope (Eclipse E100, Nikon, Japan). Muscle tissue areas were selected for 400 × imaging, and the imaging was performed to fill the entire field of view with as much tissue as possible, ensuring consistent background lighting in each photograph. After imaging, the image was analyzed using Image-Pro Plus 6.0, and the standard unit of measurement was millimeter.

### 2.3 Transcriptomic sequencing

Total RNA was extracted using the Trizol kit (Invitrogen, Carlsbad, CA, USA) according to the method provided by the manufacturer. RNA quality was assessed on an Agilent 2100 Bioanalyzer (Agilent Technologies, Palo Alto, CA, USA) and assayed using RNase-free agarose gel electrophoresis. RNA was extracted from muscle tissues, assessing mRNA quality. Then, the mRNA was purified, fragmented, and reverse transcribed into complementary DNA (cDNA). Reverse transcription to cDNA was performed with the NEBNext Ultra RNA Library Prep Kit for Illumina (NEB #7530, New England Biolabs, Ipswich, MA, USA). ligation reactions were purified with AMPure XP Beads (1.0X). Polymerase chain reaction (PCR) amplification was then performed. The cDNA library obtained was sequenced using Illumina Novaseq6000 (Ripple Gene Technology, Hangzhou, China) (23).

### 2.4 Data quality control

The raw data obtained from the sequencing platform in FASTQ format (Rawdata) contained adapter sequences and low-quality reads, making it unsuitable for direct alignment analysis. For analysis quality, the raw reads were processed to obtain clean reads (24). Quality metrics, including Q20, Q30, and GC content, were calculated. To analyze the mRNA transcripts, the following criteria were used to identify significantly differential expressed transcripts: |log2fold change | ≥ 1.5 and *p* ≤ 0.05, with *q* ≤ 1.00 to correct the *p* value calculation.

### 2.5 Differential expression analysis

FPKM (Fragments Per Kilobase of transcript per Million mapped reads) were used as normalization methods to quantify the expression levels of mRNAs. Differentially expressed RNAs were defined under the following criteria: *p* < 0.05 and an absolute fold-change > 1.

### 2.6 GO and KEGG enrichment analyses

We performed gene set enrichment analysis using software GSEA and MSigDB to identify whether a set of genes in specific GO terms KEGG pathways Reactome pathways DO terms shows significant differences in two groups. Briefly, we input gene expression matrix and rank genes by SignaltoNoise normalization method. Enrichment scores and *p* value was calculated in default parameters.

### 2.7 RT-qPCR validation

Extract total RNA from the muscles sample, take a grinding tube, add 1 ml of RNA extraction solution, add three 3 mm grinding beads, and pre-cool on ice. Take 5–20 mg of tissue and add it to the grinding tube. The grinder grinds well until there are no visible tissue blocks. Centrifuge at 12,000 rpm for 10 min at 4 °C to take the supernatant. Add 100 μl of chloroform substitute, invert the centrifuge tube for 15 s, mix well, and let stand for 3 min. Centrifuge at 12,000 rpm for 10 min at 4 °C. Transfer 400 μl of the supernatant to a new centrifuge tube, add 550 μl of isopropanol, and mix by inverting. Leave at −20 °C for 15 min. Centrifuge at 12,000 rpm at 4 °C for 10 min, and the white precipitate at the bottom of the tube is RNA. Aspirate the liquid, add 1 ml of 75% ethanol and mix to wash the pellet. Centrifuge at 12,000 rpm for 5 min at 4 °C. Repeat steps (10–11) once. Suck the liquid clean, put the centrifuge tube on the clean table and blow for 3–5 min. Add 15 μl of RNA lysis solution to dissolve RNA. Use Nanodrop 2000 to detect RNA concentration and purity: After the instrument blank is zeroed, take 2.5 μl of the RNA solution to be tested on the detection base, put down the sample arm, and use the software on the computer to start the absorbance value detection. Dilute the RNA that is too high in an appropriate ratio to a final concentration of 200 ng/μl. Reverse transcription of total RNA into cDNA. Reverse transcription reaction (20 μl reaction set, reverse transcription kit catalog number G3337) was gently mixed and centrifuged, reverse transcription program was set up, and reverse transcription was completed on a common PCR instrument for RT-qPCR primer information. Take 0.1 ml of PCR reaction plate and prepare the reaction system as follows, with 3 tubes of each reverse transcript product. After spotting the sample, the sealing film was completed with PCR sealing film and sealing instrument, and centrifugation was carried out with a microplate centrifuge. PCR amplification, which is done on a real-time PCR instrument. All samples were subjected to 3 technical replicates. All lab equipment consumables (Supplementary Table S1).

ΔΔCT method: A = CT (target gene, sample to be tested) – CT (internal standard gene, sample to be tested).

B = CT (target gene, control sample) – CT (internal standard gene, control sample).

K = A-B.

Expression fold = 2-K.

## 3 Results and analysis

### 3.1 RNA-Seq data analysis

A total of 15 cDNA libraries were generated. As shown in Table 1, the muscle transcriptome produced ~850 million high-quality reads (56,533,511.2 reads per library). The GC content ranged from 50.89 to 52.27%, while the Q20 and Q30 scores were between 98.50–98.65% and 95.42–95.87%, respectively. Furthermore, over 91.41% of the clean reads were aligned with the reference genome.

**Table 1:** Overall detection of mRNA sequencing data.

**Samples**	**Raw data**	**Clean data**	**Q20**	**Q30**	**GC content**	**Mapped reads**
Gb-1	52,600,700	51,228,464 (97.39%)	98.59%	95.69%	51.56%	46,831,680 (91.41%)
Gb-2	60,924,228	59,514,598 (97.69%)	98.64%	95.84%	51.35%	54,126,030 (90.95%)
Gb-3	59,821,814	58,231,030 (97.34%)	98.58%	95.67%	52.78%	53,132,286 (91.24%)
Gb-4	62,928,188	61,088,904 (97.08%)	98.65%	95.87%	52.15%	55,202,427 (90.36%)
Gb-5	61,793,134	60,379,646 (97.71%)	98.64%	95.85%	51.54%	55,184,736 (91.40%)
Gf-1	61,411,500	59,943,692 (97.61%)	98.61%	95.73%	50.92%	54,954,663 (91.68%)
Gf-2	55,196,862	53,930,098 (97.71%)	98.64%	95.85%	51.04%	49,656,683 (92.08%)
Gf-3	51,898,192	50,491,302 (97.29%)	98.50%	95.42%	51.83%	46,195,189 (91.49%)
Gf-4	44,219,856	43,104,312 (97.48%)	98.64%	95.83%	51.83%	39,584,418 (91.83%)
Gf-5	54,530,348	53,223,174 (97.6%)	98.55%	95.56%	51.81%	48,679,195 (91.46%)
Gg-1	58,889,028	57,464,686 (97.58%)	98.56%	95.60%	52.27%	52,788,024 (91.86%)
Gg-2	60,469,756	58,982,602 (97.54%)	98.62%	95.78%	51.02%	54,006,312 (91.56%)
Gg-3	54,807,072	53,441,718 (97.59%)	98.57%	95.61%	51.64%	49,034,062 (91.75%)
Gg-4	53,987,080	52,746,006 (97.70%)	98.65%	95.86%	51.11%	48,074,178 (91.14%)
Gg-5	54,524,910	53,203,350 (97.58%)	98.63%	95.80%	50.89%	48,404,886 (90.98%)

Note: Samples: Sample name.

Raw Data: Original sequencing data.

Clean Data: Filter the data.

Q20 Proportion of bases with quality value greater than or equal to 20.

Q30 Proportion of bases with quality value greater than or equal to 30.

GC content: Calculate the percentage of the total number of bases G and C to the total number of bases.

Mapped reads: Comparing reads to the genome.

### 3.2 Morphological observation of muscle tissues

**Figure 2:**
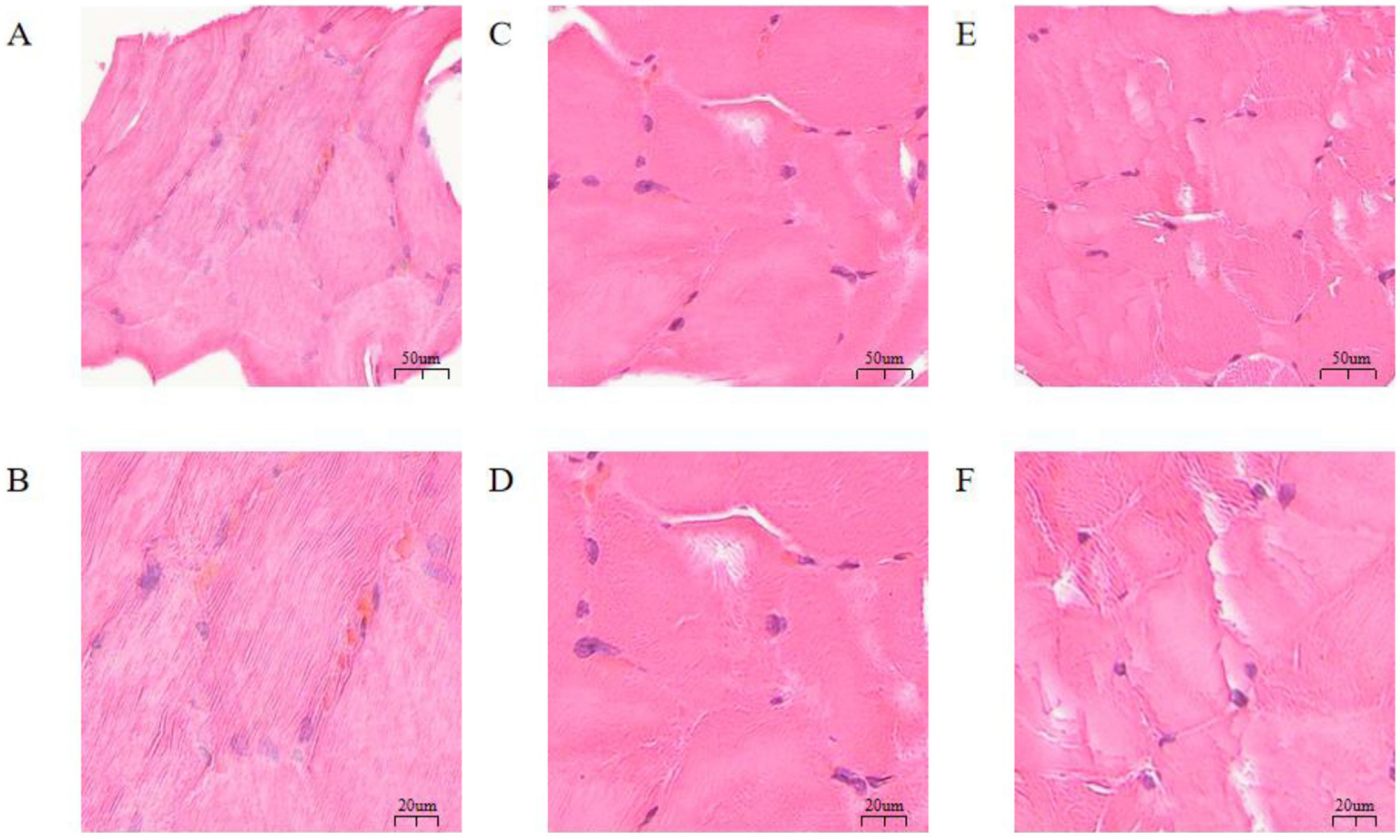
Morphological Observation of Muscle Tissues from the Gb, Gf, and Gg Groups. **(A)** 20× magnification of the tissue section from the Gb group; **(B)** 50× magnification of the tissue section from the Gb group; **(C)** 20× magnification of the tissue section from the Gf group; **(D)** 50× magnification of the tissue section from the Gf group; **(E)** 20× magnification of the tissue section from the Gg group; and **(F)** 50× magnification of the tissue section from the Gg group.

**Figure 3:**
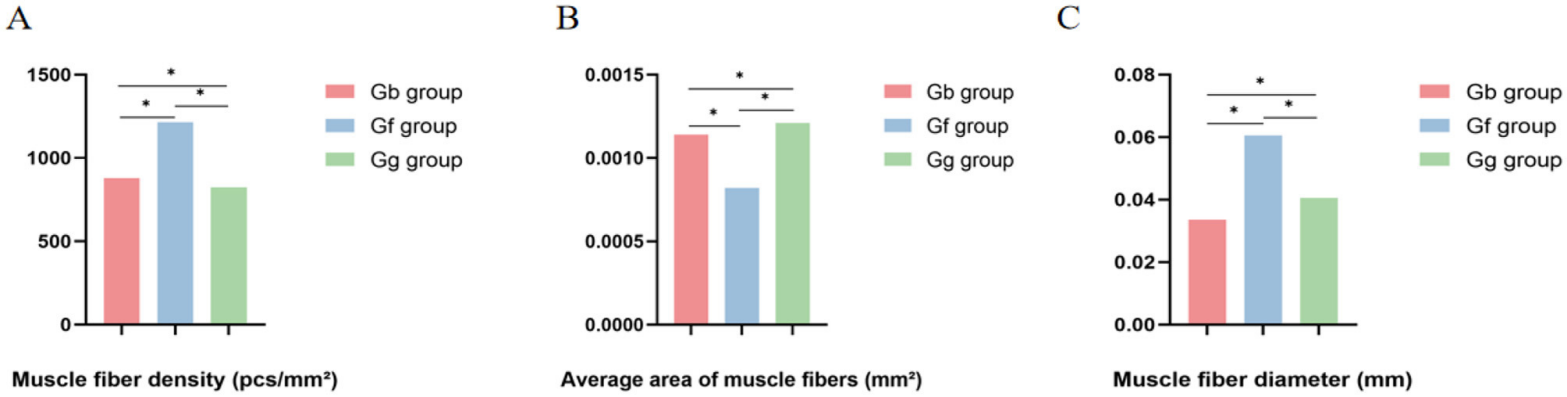
Differences related to muscle fiber density, average area of fibers and muscle fiber diameter in Gb, Gf, and Gg groups. Differences related to muscle fiber density in Gb, Gf, and Gg groups; Differences related to average area of fibers in Gb, Gf, and Gg groups; **(C)** Differences related to muscle fiber diameter in Gb, Gf, and Gg groups.

Histological examination of the muscle tissues using H&E staining revealed varying developmental characteristics, as shown in Figure 2. Indicators such as fiber diameter, density, and area were used to reflect the muscle development in different regions. As shown in Figure 3, the muscle fiber density in the Gf group was higher than that in both the Gb and Gg groups, with significant differences between the groups (*p* < 0.05), the average area of muscle fibers in the Gg group was higher than that in the Gb and Gf groups, with significant differences between the groups (*p* < 0.05), and the muscle fiber diameter in the Gf group was higher than that in the Gb and Gg groups, with significant differences between the groups (*p* < 0.05) (see Supplementary Table S2). The muscle fiber nuclei were evenly distributed, and the fibers appeared nearly elliptical with multiple nuclei located near the cell membrane. Both the cytoplasm and the nuclei exhibited clear staining. The muscle fibers contained multiple fiber bundles.

### 3.3 Sample correlation analysis

**Figure 4:**
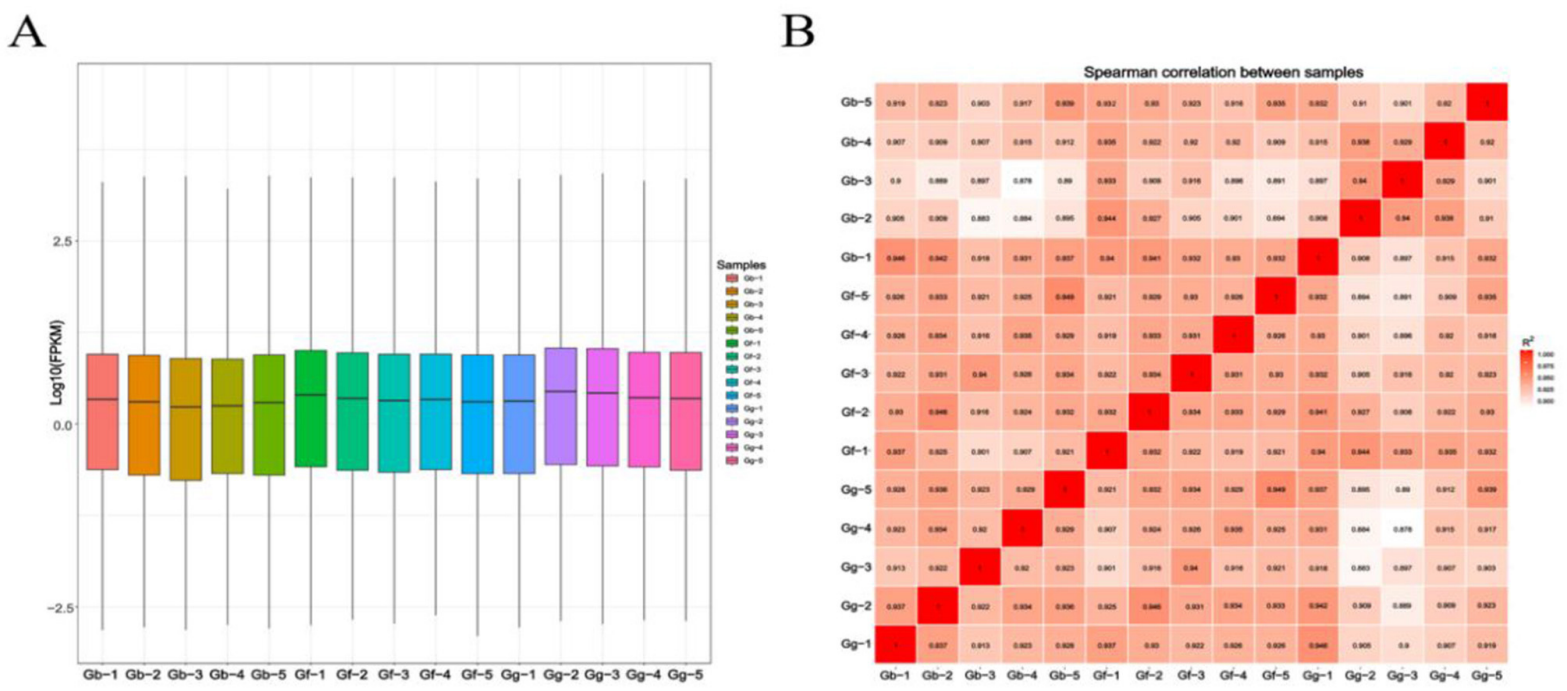
Boxplot of Expression Levels and Correlation Heatmap for the Gb, Gf, and Gg Groups. **(A)** Boxplot of expression levels across the samples; and **(B)** Correlation heatmap for the groups.

As shown in Figure 4A, the Gg group exhibited the highest expression levels, while the Gb group showed the lowest. There was little difference in the expression level among different samples, and the expression level was generally consistent among individual samples. Figure 4B illustrates a similar trend in sample correlation between the groups.

### 3.4 Differential expression analysis

**Figure 5:**
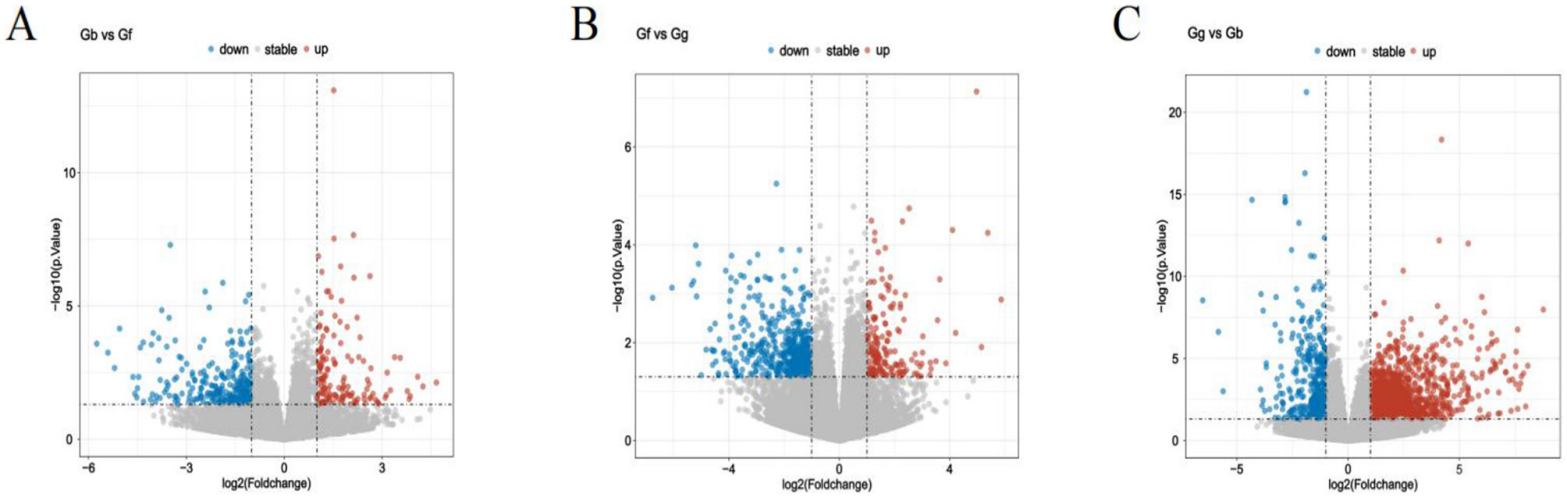
Volcano Plots of DEGs in the Gb, Gf, and Gg Groups. **(A)** Volcano plot for the Gb and Gf groups; **(B)** Volcano plot for the Gf and Gg groups; and **(C)** Volcano plot for the Gg and Gb groups. Note: In Figures, “up” and “down” represent upregulated and downregulated genes, respectively.

As depicted in Figure 5A, 426 DEGs were identified between the Gb and Gf groups, including *PEBP4, PTP4A3, PDLIM7*, and *MYL6B*. Of these, 147 genes were upregulated, and 279 were downregulated. As illustrated in Figure 5B, 1,762 DEGs were identified between the Gf and Gg groups, including *MYBPH, SLC39A8, EMX2*, and *GRB7*. Among these, 1,391 genes were upregulated, and 371 were downregulated. Additionally, as shown in Figure 5C, 644 DEGs were identified between the Gg and Gb groups, including *HOXD9, TBX1, LDHA*, and *PKM*. Of these, 172 genes were upregulated, and 472 were downregulated (see Supplementary Table S3).

**Figure 6:**
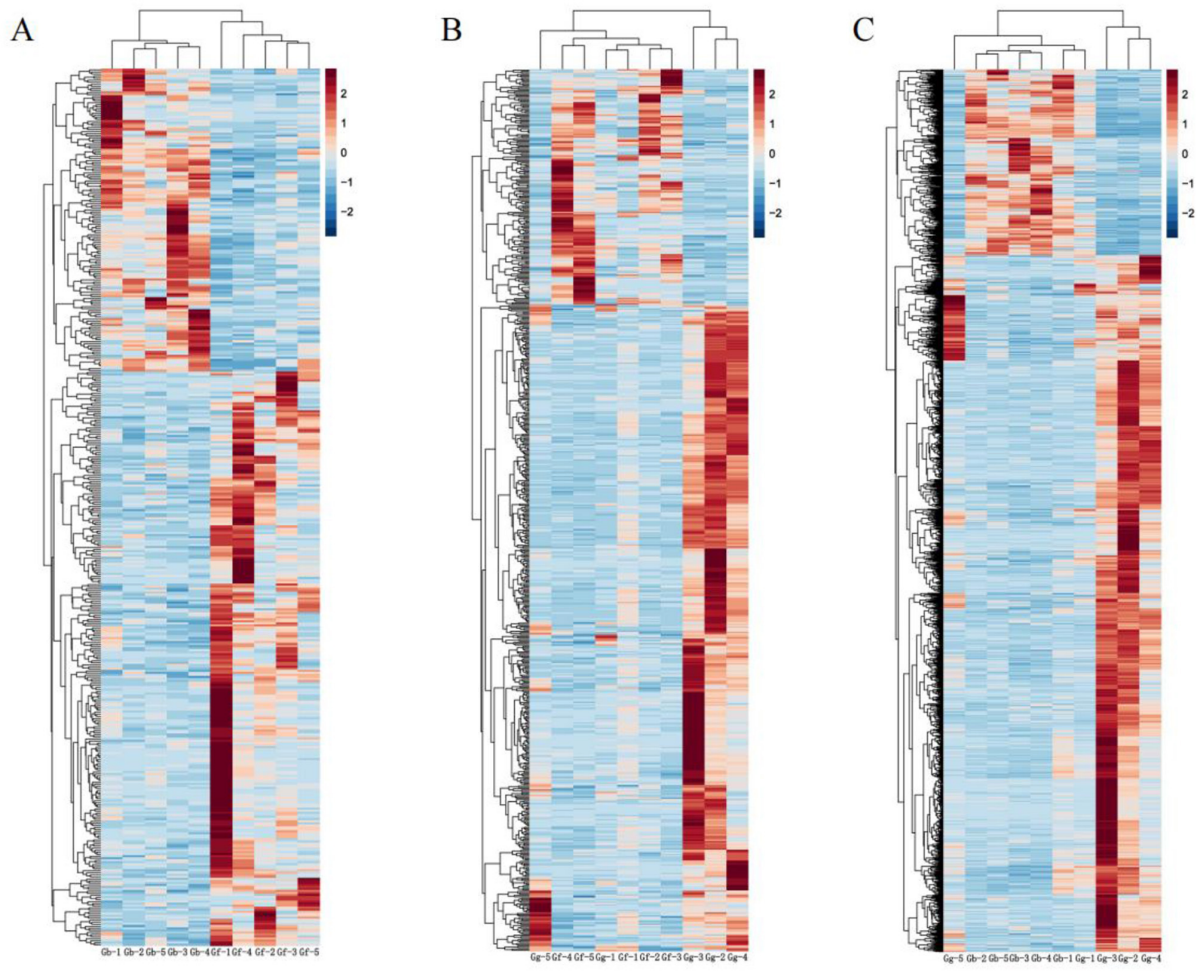
Clustering Analysis of DEGs in the Gb, Gf, and Gg Groups. **(A)** Clustering analysis for the Gb and Gf groups; **(B)** Clustering analysis for the Gf and Gg groups; and **(C)** Clustering analysis for the Gg and Gb groups. Note: In Figures, the *x*-axis denotes individual samples, and the *y*-axis represents expression levels. The color gradient from blue to red indicates increasing upregulation.

Clustering analysis results are displayed in Figures 6A–C. The muscle tissues from different regions of Kazakh horses exhibited high reproducibility, revealing significant differences between the groups.

### 3.5 GO functional annotation and KEGG enrichment analysis of DEGs

The functional enrichment of DEGs was categorized into three types: cellular component (CC), molecular function (MF), and biological process (BP).

**Figure 7:**
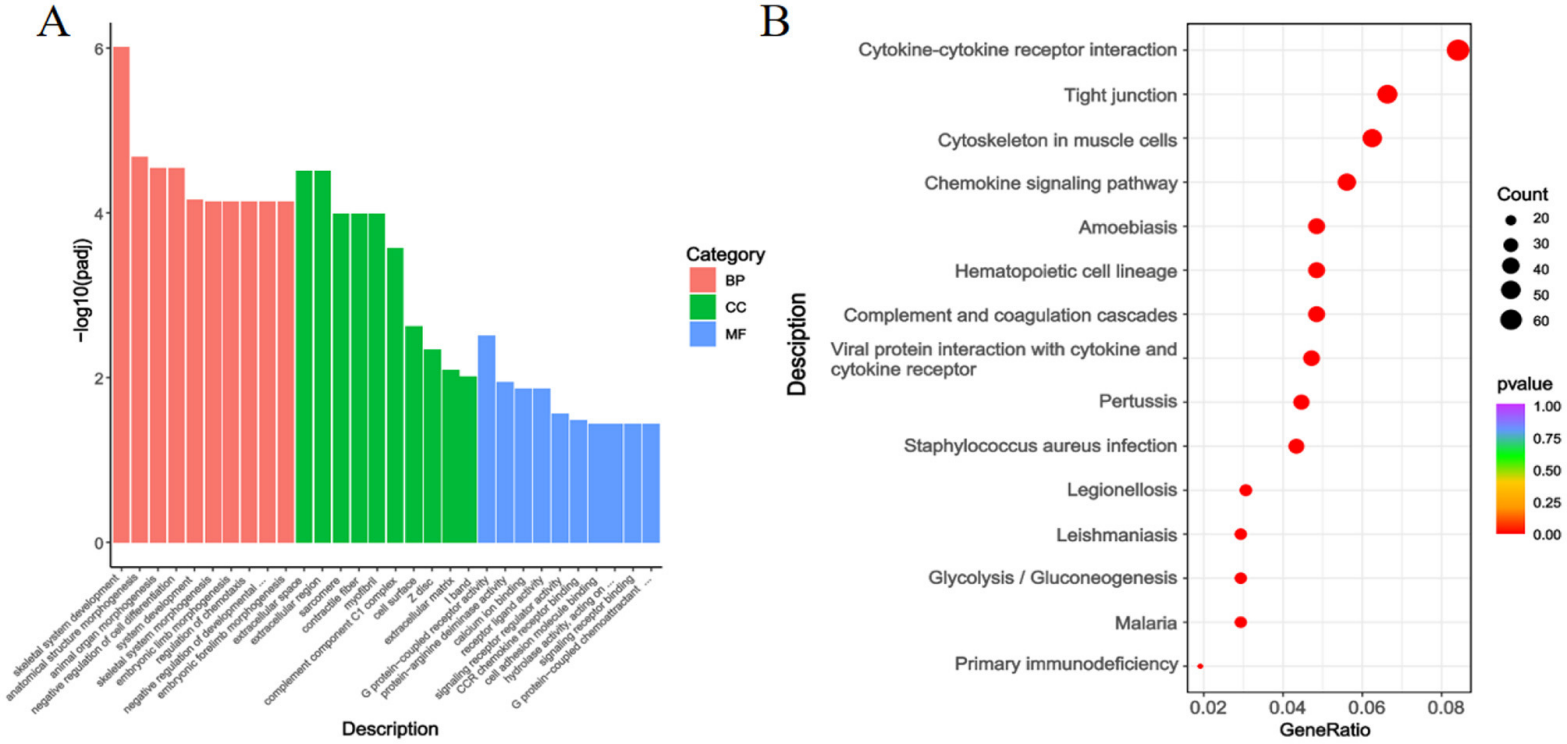
GO Annotation and KEGG Enrichment Analysis of DEGs in the Gb and Gf, Groups. **(A)** GO annotation of DEGs between the Gb and Gf groups; **(B)** KEGG enrichment analysis of DEGs between the Gb and Gf groups.

As shown in Figure 7A, the GO annotation results for the Gb and Gf groups indicated that DEGs were mainly enriched in terms related to Skeletal System Development (BP), Anatomical Structure Morphogenesis (BP), Extracellular Space (CC), Extracellular Region (CC), G Protein-Coupled Receptor Activity (MF), and Protein-Arginine Deiminase Activity (MF).

Figure 7B presents the KEGG enrichment analysis for the Gb and Gf groups. DEGs were primarily enriched in pathways such as Cytoskeleton in Muscle Cells, Cytokine-Cytokine Receptor Interaction, and Motor Proteins.

**Figure 8:**
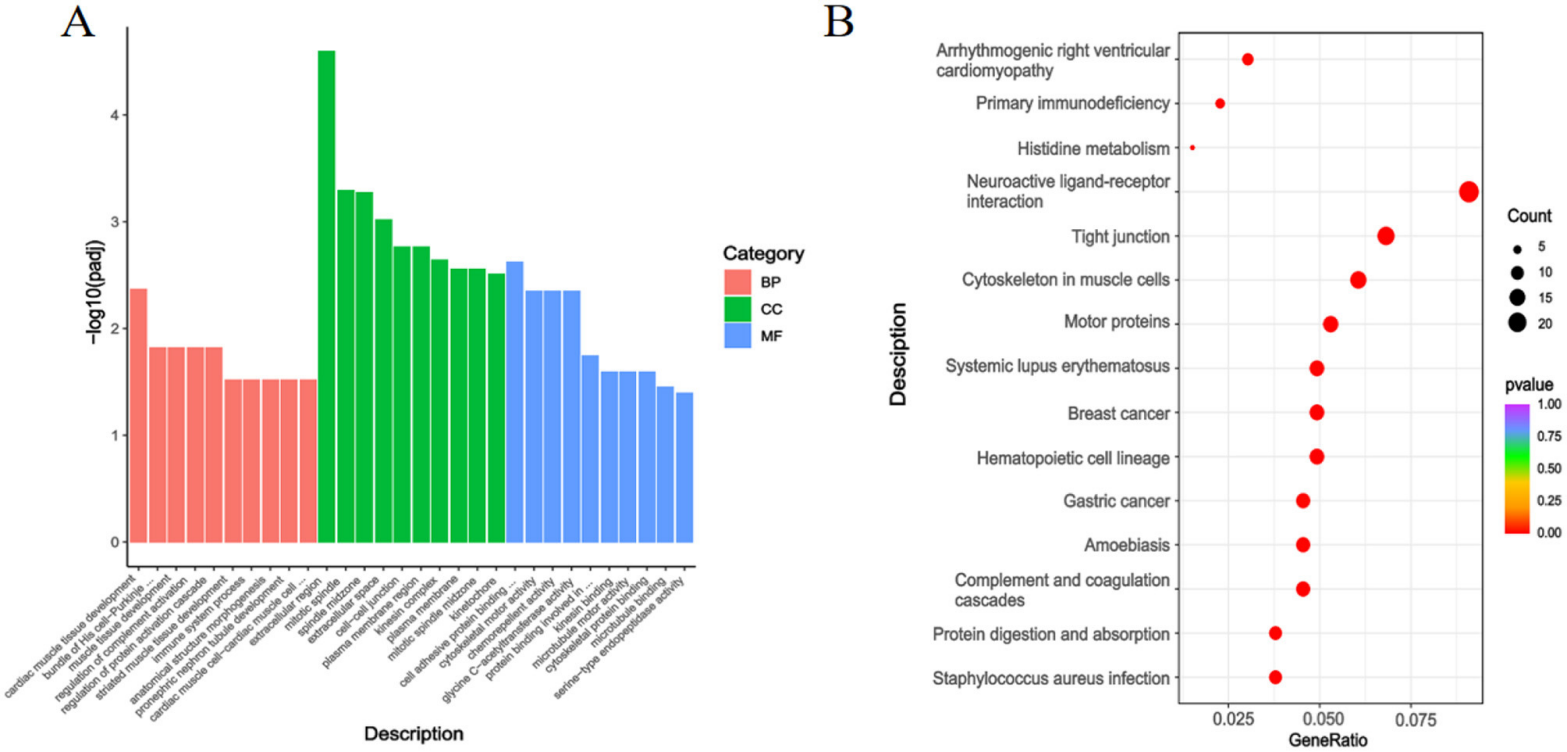
GO Annotation and KEGG Enrichment Analysis of DEGs in the Gf and Gg, Groups. **(A)** GO annotation of DEGs between the Gf and Gg groups; **(B)** KEGG enrichment analysis of DEGs between the Gf and Gg groups.

The GO annotation results in Figure 8A for the Gf and Gg groups demonstrated that DEGs were mainly involved in processes such as Cardiac Muscle Tissue Development (BP), Muscle Tissue Development (BP), Extracellular Region (CC), Mitotic Spindle (CC), Cytokine Receptor Activity (MF), and Cytoskeletal Motor Activity (MF).

Figure 8B displays the KEGG enrichment analysis for the Gf and Gg groups. DEGs were enriched in pathways including Neuroactive Ligand-Receptor Interaction, Tight Junction, and Cytoskeleton in Muscle Cells.

**Figure 9:**
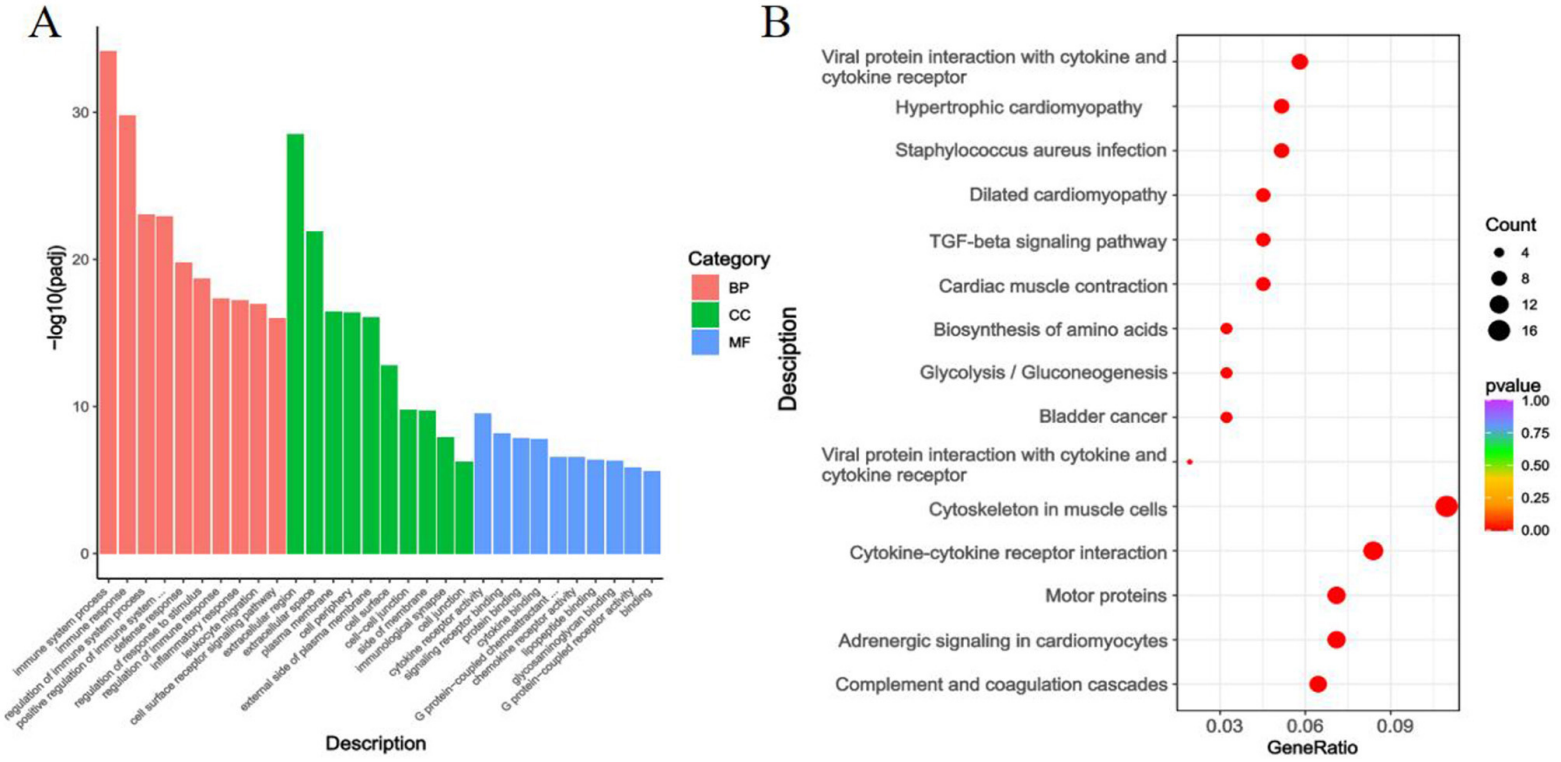
GO Annotation and KEGG Enrichment Analysis of DEGs in the Gg and Gb Groups. **(A)** GO annotation of DEGs between the Gg and Gb groups; **(B)** KEGG enrichment analysis of DEGs between the Gg and Gb groups. Note: In Figures 7A, 8A, and 9A, the *x*-axis represents each DEG, with red indicating biological process (BP), green denoting cellular component (CC), and blue signifying molecular function (MF). Figures 7B, 8B, and 9B illustrate the top 15 pathways with the lowest *Q*-values. The *y*-axis shows the pathway names, and the *x*-axis represents the gene ratio. The “count” denotes the quantity, and the color gradient from blue to red indicates decreasing *Q*-values.

As depicted in Figure 9A, the GO annotation results for the Gg and Gb groups showed that DEGs were primarily enriched in pathways related to Immune System Process (BP), Immune Response (BP), Extracellular Region (CC), Extracellular Space (CC), Cytokine Receptor Activity (MF), and Signaling Receptor Binding (MF).

Figure 9B exhibits the KEGG enrichment analysis for the Gg and Gb groups. DEGs were predominantly enriched in pathways like Cytokine-Cytokine Receptor Interaction, Tight Junction, and Transcriptional Misregulation in Cancer (see Supplementary Table S4).

### 3.6 RT-qPCR validation

**Figure 10:**
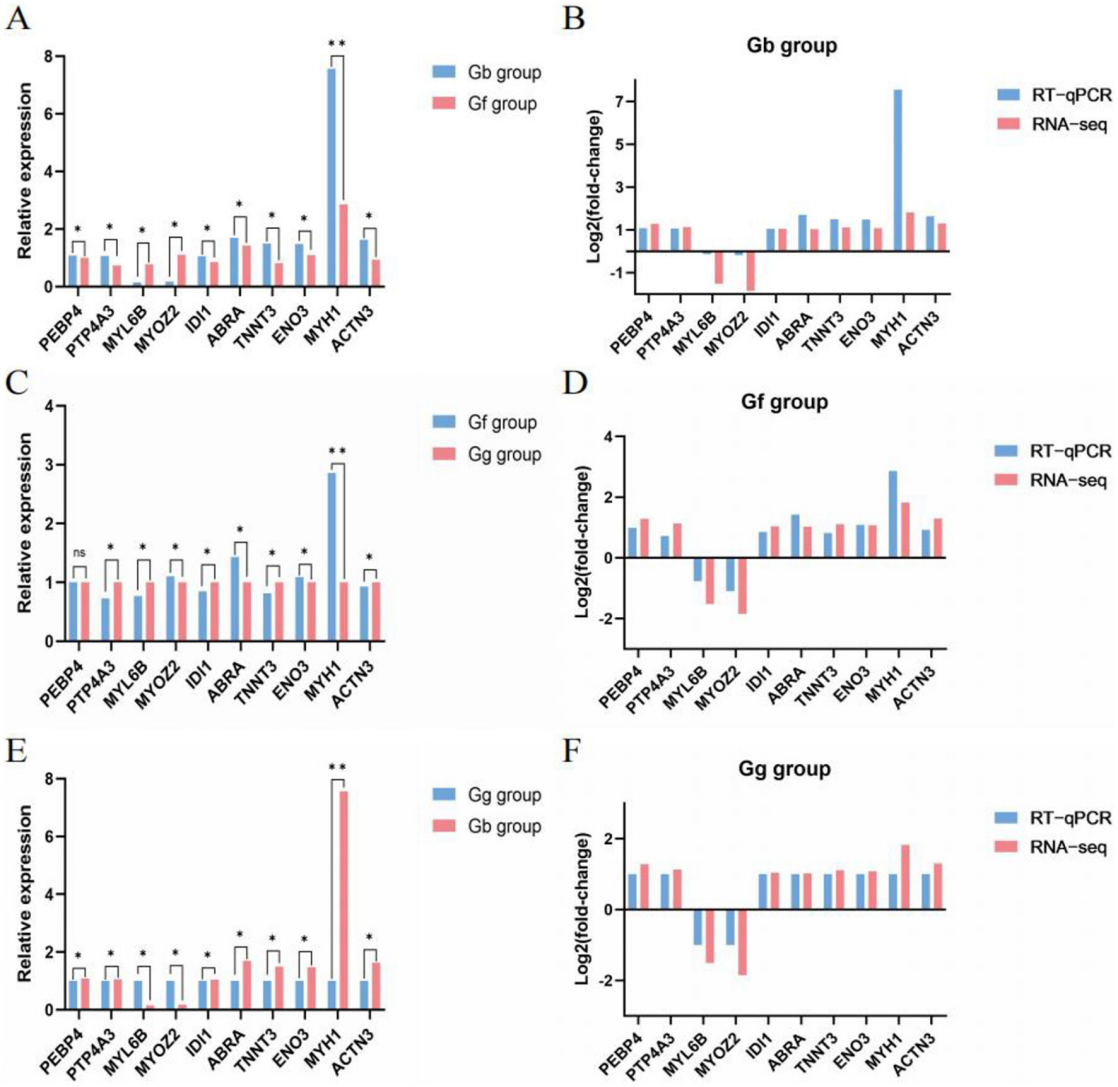
RT-qPCR Validation. **(A)** RT-qPCR of differential expression between the Gb and Gf groups; **(B)** Log2-fold change in Gb group differential genes between RNA sequencing (RNA-seq) and reverse transcription quantitative polymerase chain reaction (RT-qPCR) (Log2FC); **(C)** RT-qPCR of differential expression between the Gf and Gg groups; **(D)** Log2-fold change in Gf group differential genes between RNA sequencing (RNA-seq) and reverse transcription quantitative polymerase chain reaction (RT-qPCR) (Log2FC); **(E)** RT-qPCR of differential expression between the Gg and Gb groups; **(F)** Log2-fold change in Gg group differential genes between RNA sequencing (RNA-seq) and reverse transcription quantitative polymerase chain reaction (RT-qPCR) (Log2FC); Note: In the figure, “^*^” indicates a significant difference between the two groups (*p* < 0.05); “^**^” indicates a highly significant difference between the two groups (*p* < 0.01); “ns” indicates that there is no difference between the two groups.

In order to validate the accuracy of the transcriptome sequencing data, this study randomly selected the following DEGs for RT-qPCR: *PEBP4, PTP4A3, IDI1, ABRA, TNNT3, ENO3, MYH1, TNNI2, PGAM2*, and *ACTN3*. As illustrated in Figure 10, the expression levels of *PEBP4, PTP4A3, IDI1, ABRA, TNNT3, ENO3, TNNI2, PGAM2*, and *ACTN3* were significantly upregulated (*p* < 0.05), while *MYH1* showed a highly significant increase (*p* < 0.01). The expression trends of RT-qPCR and RNA-seq results were consistent, confirming the reliability and authenticity of the sequencing data and expression profiles. Therefore, they can be used for subsequent analysis.

## 4 Discussion

Mammalian muscles encompass skeletal muscle, smooth muscle, and cardiac muscle, which differ significantly in both function and morphology (25). Skeletal muscle development is a complex biological process that involves myoblast proliferation, differentiation, myotube fusion, and muscle fiber formation (26). Skeletal muscle predominantly relies on aerobic metabolism, requiring oxygen to sustain metabolic activity. Consequently, skeletal muscle contains abundant myoglobin and capillaries, giving it a characteristic red color. The pH of skeletal muscle directly impacts meat quality. Following slaughter, skeletal muscle continues metabolic activities, primarily aerobic metabolism. This results in a gradual decline in pH, producing less lactic acid and leading to better meat quality (27).

The quality of horse meat is influenced by various factors, including breed, age, gender, and anatomical location. Wang et al. (21) identified genes such as *RYR3* and *MYH6* as key regulators of muscle function within both fast and slow muscle fibers in male and female Kazakh horses, potentially affecting skeletal muscle fiber composition and enhancing meat quality. Jia (28) observed that the pH of Mongolian horse cervical muscles was the highest, followed by shoulder and forelimb muscles, which were significantly higher than that of the thoracic and lumbar muscles. The lowest pH was found in the longissimus dorsi muscles. The triceps brachii and gluteus medius muscles exhibited a high proportion of slow-twitch muscle fibers (>70%), which is desirable for high-quality meat. Furthermore, muscles in the head and neck region were enriched in lipid and amino acid metabolism pathways, which might contribute to a unique flavor profile, offering potential for meat quality development.

This study performed transcriptomic analysis on muscle tissues from the Gb, Gf, and Gg groups of Kazakh horses. KEGG pathway enrichment analysis revealed that DEGs were primarily enriched in muscle-related signaling pathways, including Cytoskeleton in Muscle Cells, Cytokine-Cytokine Receptor Interaction, and Motor Proteins. Claeyssen et al. (29) demonstrated that Desmin maintains muscle architecture and contraction efficiency. Desmin is modified by O-GlcNAcylation. Following Thiamet G treatment, O-GlcNAcylation increases, while Desmin remains unchanged. The reduced distribution of Desmin to the cytoskeleton suggests that O-GlcNAcylation may be involved in cytoskeletal remodeling. Muscle development and structural integrity rely on myoblast differentiation and myofiber formation. Disruptions in these processes can compromise muscle function, which is a primary factor in the pathogenesis of skeletal muscle disorders (30–33).

Fu et al. (34) found that coordinated interaction between the cytoskeleton and mitochondria is vital for muscle development and function. *PRR33* regulates this cytoskeleton-mitochondria interplay by interacting with Desmin, with dysregulation potentially resulting in Desmin accumulation and mitochondrial dysfunction in muscle fibers. The Cytokine-Cytokine Receptor Interaction pathway plays a fundamental role in intercellular communication and signaling. It regulates various physiological processes, governing critical physiological processes such as cell growth, differentiation, immune activation, inflammation, and hematopoiesis (35, 36). Consequently, skeletal muscle growth, development, and structural function are intrinsically linked to meat quality. Huang et al. (37) Fat mass- and obesity-associated (FTO) genes play an important role in promoting myoblast differentiation in chickens and are significantly enriched in pathways such as the Cytokine-Cytokine Receptor Interaction. Mice deficient in FTO exhibit immediate postnatal growth retardation, shorter body lengths, lower body weights, and lower bone mineral densities, which severely affects skeletal muscle development and leads to increased muscle tenderness (38). For instance, Wang et al. (39) conducted transcriptomic analysis on Landrace longissimus dorsi muscles and liver, revealing that DEGs in longissimus dorsi tissue were mainly involved in growth, development, and immune regulation, with significant enrichment in the Cytokine-Cytokine Receptor Interaction pathway, consistent with this study. Zhang et al. (40) investigated the growth performance of broiler chickens, where transcriptomic analysis of breast muscle revealed that *RAC2* might regulate cell proliferation via pathways including Cytokine-Cytokine Receptor Interaction and PAKs/MAPK8, thereby influencing chicken growth and meat quality.

Numerous studies have examined the genetic underpinnings of meat quality through gene expression and co-expression analyses across different species (41–43). Zhang et al. (44) reported that DEGs such as *TNNI1, ALDH2, CDC37*, and *ATP8* are predominantly enriched in pathways related to muscle fiber structure, fatty acid metabolism, amino acid processing, ion channel binding, protein processing, and energy production. These DEGs exhibit region-specific expression patterns in bovine muscle tissues, playing pivotal roles in the regulation of beef quality and muscle fiber development. In this study, transcriptomic sequencing was employed to analyze the gene expression profiles of muscle tissues from various anatomical regions of Kazakh horses. The analysis revealed region-specific gene modules, including *TPM1, TNNI2, ACTN3, CSF1R*, and *MYH8*. *TPM1*, a member of the Tropomyosin (Tm) family, is an evolutionarily conserved Actin-Binding Protein (ABP) involved in the contraction systems of striated and smooth muscles, as well as the development and stability of muscle cell cytoskeletons. Chai et al. (45) conducted differential analysis of muscle tissues from various anatomical regions in Texas donkeys using transcriptome sequencing, indicating that genes within the Tropomyosin family might influence meat tenderness and are involved in modulating muscle fiber types and glucose metabolism, ultimately improving meat quality. *TNNI2*, a member of the Troponin I gene family, is predominantly expressed in fast-twitch skeletal muscle fibers and is a critical regulatory protein for cardiac muscle contraction. It plays a fundamental role in both muscle contraction and relaxation. Kumar et al. (46) demonstrated that hub genes such as *TNNI2, TNNT3*, and *ACTN3* co-express in the longissimus thoracis muscle of goats, where they are implicated in regulating muscle fiber types and contraction dynamics. Skeletal muscle fibers exhibit structural changes in response to stimulation, which are associated with intramuscular fat synthesis in goats. These changes influence both meat quality and lipid metabolism. Li et al. (47) further supported these findings through a transcriptomic comparison of the longissimus dorsi and soleus muscles in Landrace pigs, revealing that DEGs like *TNNT1, TNNC1*, and *SRPK3* are enriched in biological processes associated with muscle fiber differentiation and contraction. These genes facilitate the conversion of slow-twitch muscle fibers into fast-twitch fibers and modulate *GLUT4* expression, playing a crucial role in the regulation of pork meat quality.

In this study, we sequenced the transcriptome of muscle tissues of male kazakh horse muscles from various anatomical locations. The results of this experiment can be generalized to female kazakh horse, and the group is actively completing experiments on muscle tissues of female kazakh horse muscles from various anatomical locations.

## 5 Conclusions

This study employed transcriptomic sequencing to analyze muscle tissues from diverse anatomical locations in Kazakh horses. The findings demonstrated that DEGs such as *TPM1, TNNI2, ACTN3, CSF1R*, and *MYH8* play active roles in key pathways related to skeletal muscle growth, development, and metabolism, including Cytoskeleton in Muscle Cells, Cytokine-Cytokine Receptor Interaction, and Motor Proteins. Consequently, these genes regulate muscle development in Kazakh horses, modulate muscle fiber types and tenderness, and promote meat quality and palatability.

## Data Availability

The datasets presented in this study can be found in online repositories. The names of the repository/repositories and accession number(s) can be found in the article/Supplementary material.
